# Segmentation of thyroid glands and nodules in ultrasound images using the improved U-Net architecture

**DOI:** 10.1186/s12880-023-01011-8

**Published:** 2023-04-14

**Authors:** Tianlei Zheng, Hang Qin, Yingying Cui, Rong Wang, Weiguo Zhao, Shijin Zhang, Shi Geng, Lei Zhao

**Affiliations:** 1 School of Information and Control Engineering, China University of Mining and Technology, Xuzhou, 221116 China; 2 Artificial Intelligence Unit, Department of Medical Equipment Management, Affiliated Hospital of Xuzhou Medical University, Xuzhou, 221004 China; 3 Department of Medical Equipment Management, Nanjing First Hospital, Nanjing, 221000 China; 4 Department of Pathology, Affiliated Hospital of Xuzhou Medical University, Xuzhou, 221004 China; 5 Department of Ultrasound Medicine, Affiliated Hospital of Xuzhou Medical University, Xuzhou, 221004 China

**Keywords:** Convolutional neural network, Deep learning, Ultrasound images, Semantic segmentation, Thyroid nodule, U-Net

## Abstract

**Background:**

Identifying thyroid nodules’ boundaries is crucial for making an accurate clinical assessment. However, manual segmentation is time-consuming. This paper utilized U-Net and its improved methods to automatically segment thyroid nodules and glands.

**Methods:**

The 5822 ultrasound images used in the experiment came from two centers, 4658 images were used as the training dataset, and 1164 images were used as the independent mixed test dataset finally. Based on U-Net, deformable-pyramid split-attention residual U-Net (DSRU-Net) by introducing ResNeSt block, atrous spatial pyramid pooling, and deformable convolution v3 was proposed. This method combined context information and extracts features of interest better, and had advantages in segmenting nodules and glands of different shapes and sizes.

**Results:**

DSRU-Net obtained 85.8% mean Intersection over Union, 92.5% mean dice coefficient and 94.1% nodule dice coefficient, which were increased by 1.8%, 1.3% and 1.9% compared with U-Net.

**Conclusions:**

Our method is more capable of identifying and segmenting glands and nodules than the original method, as shown by the results of correlational studies.

## Background

In recent years, the incidence of thyroid cancer has been increasing [1, 2]. Researches have shown that early identification of thyroid nodules and prevention of calcification can significantly lower thyroid cancer mortality [3, 4]. MRI, CT, and Ultrasound (US) are some of the conventional methods used to examine thyroid nodules [5–8]; among the various methods, US is the preferred method of thyroid examination due to its lack of radiation, convenience, real-time performance and high resolution [8].

The US examination imaging and diagnosis are conducted independently by only one physician, in contrast to MRI and CT examinations. Otherwise, the physician’s level of experience, status, and sentiment would influence the accuracy of the diagnosis, which would result in greater subjectivity in the US diagnosis [7].

Because nodules within the thyroid both size and location vary significantly among groups, ultrasound images are susceptible to spots and echoes [9]. These noises affect the visual quality of the images, and how to accurately find the fuzzy boundary between thyroid parenchyma and nodules is a challenge for young physicians [5, 7, 10–12].

Segmentation detects the region of interest of an image so as to accurately divide the boundary between the thyroid parenchyma and nodules. The precise segmentation of thyroid nodules has become an indispensable step for research because it can effectively determine the size and location of nodules, which doctors may use to issue diagnostic reports and develop treatment plans [8]. Developing automated segmentation methods can effectively lessen the reliance on physicians' diagnostic expertise because manual segmentation is tedious and time-consuming [13].

Traditional machine learning methods and deep learning methods have been widely used in thyroid segmentation [14–17]. Selvathi and Sharnitha [7] used the Extreme Learning Machine to segment the thyroid, and it achieved results that were obviously superior to those of the support vector machine (SVM). However, as a traditional machine learning method, it is slightly less robust and difficult to adapt to more complex and noisy situations. Ma et al. [18] automatically segmented ultrasonic images based on a convolutional neural network (CNN), compared the CNN with conventional segmentation methods, such as SVM and radial basis function Neural Network, and found that the CNN provided better performance. However, CNN cannot correctly segment some thyroid nodules with very complex and similar backgrounds. Kumar et al. [6] applied a CNN to segment both thyroid glands, nodules and cystic components. However, due to the complexity of the task and the small number of samples caused by task constraints, their segmentation effect was slightly worse than that of simple segmentation of glands and nodules.

In the domain of deep convolutional networks, U-Net and its improved method are widely applied to medical tasks [19], including wounds [20], colorectal cancer [21], and thyroid segmentation [22–24]. However, U-Net still has room for improvement in thyroid segmentation. One improvement opportunity is the imperfect segmentation of edges and tiny nodules caused by the insufficient extraction of features from high-resolution data [25], and another is the slightly flawed segmentation of large and irregular targets. These problems can be mitigated by the introduction of ResNeSt block, atrous spatial pyramid pooling (ASPP) and deformable convolution (DC) v3. ResNeSt is a general CNN model that has a good segmentation effect on both the ADE20K and Cityscapes datasets [26]. The stronger feature extraction ability of ResNeSt block can optimize the segmentation of small targets. ASPP presented in DeepLab v3 has the ability to extract context information, and its effect is also verified using VOC 2012 [27–29]. Its larger and more numerous sights may alleviate the U-Net’s problem of extracting large target information. DC v3 can freely select the processing area according to the offset, which makes it have a larger and more targeted field of view [30]. DC v3 has good adaptability to special shape targets and deformation caused by different angles, and is suitable for segmentation of irregular glands and nodules caused by physician's manipulation or their own characteristics.

In this paper, we present a method called deformable-pyramid split-attention residual U-Net (DSRU-Net) to improve U-Net with ResNeSt block, ASPP and DC v3 for thyroid segmentation tasks. To verify the effectiveness of our model, we obtained 5822 thyroid ultrasound images via collection and screening and labeled the glands and nodules in the images in combination with pathological training and testing. We adopt the assessment criteria including the dice coefficient to compare 6 semantic segmentation networks including U-Net and DSRU-Net. The experiment shows that DSRU-Net, which obtains a 92.5% average dice coefficient and a 94.1% nodule dice coefficient, has the optimal segmentation effect on this dataset.

## Materials and methods

### Image acquisition and preprocessing

A total of 76,496 ultrasonic images of 5021 patients from the Affiliated Hospital of Xuzhou Medical University (AHXMU) and Nanjing First Hospital (NFH) from 2012 to 2018 were retrospectively analyzed. The dataset contained benign samples, inflammatory nodules, cystic nodules, and tumor nodules as well as malignant samples, including papillary carcinoma and follicular carcinoma. We handled the images with various machines and different diagnostic specialists, as well as the corresponding pathological reports. 5822 Ultrasound images were collected following the screening of these pathological reports by three associate chief physicians. Physicians screened the images in accordance with three main criteria: the first was whether the image quality was qualified; the second was whether there were thyroid glands and lesions in the image; and the third was whether the lesion in the image was consistent with the pathological diagnosis result. Following the screening, images were manually delineated with LabelMe software, and pathological reports were used to accurately sketch and record thyroid nodules and glands. A mask matrix with a background of 0, glands of 1, and nodules of 2 was generated from the sketched images. Then, in order to support the learning and training of the network model, images and masks were incorporated into a semantic segmentation network model for feature extraction, amplification, and property recognition.

### Software and hardware environment

Python 3.6.8 was used as the programming language, and PyTorch 1.5.1 was used as the deep learning toolkit [31]. Cuda 10.0 was used for the parallel computing framework, and CUDNN 7.5.0 was used to accelerate the deep neural network computing. A computer with two Intel(R) Xeon(R) Gold 6230 CPUs, two NVIDIA Quadro GV100 (32 GB of memory) GPUs and 384 GB of memory was used as the hardware environment of the experiment.

### Model architecture

**Fig. 1:**
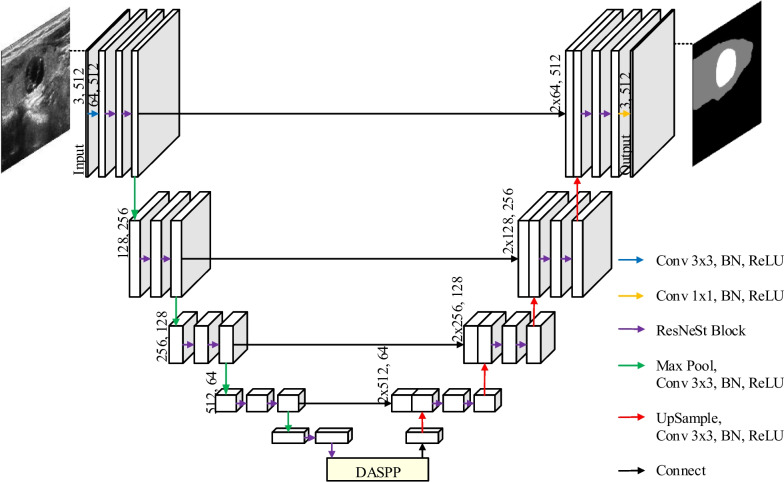
The overall architecture of DSRU-Net

DSRU-Net is mainly composed of Split-Attention Residual U-Net (SRU-Net) based on U-Net and ResNeSt block, and DASPP based on ASPP and DC v3. The overall architecture of DSRU-Net is shown in Fig. 1. As an improved model based on U-Net, DSRU-Net firstly introduces ResNeSt block with better feature extraction tendency in encoder and decoder to enhance the feature extraction capability. Then ASPP is introduced between encoder and decoder which can extract multi-scale features to improve the segmentation ability of different size targets. Finally, DC v3 which can adapt to different shape features is introduced in ASPP to improve the adaptability of the model to special shape targets.

1 $$\begin{array}{*{20}l} {f_{1} , f_{2} , f_{3} , f_{4} , f_{5} = {\text{Encoder}}\left( x \right)} \\ \end{array}$$

2 $$\begin{array}{*{20}l} {\hat{y} = {\text{Decoder}}\left( {f_{1} , f_{2} , f_{3} , f_{4} , {\text{DASPP}}\left( {f_{5} } \right)} \right)} \\ \end{array}$$

#### SRU-Net

ResNeSt is an excellent general backbone that includes the split-transform-merge inherited from GoogleNet and ResNeXt [32, 33] and the attention mechanism inspired by SENet and SKNet [34, 35]. Therefore, we make an effort to introduce ResNeSt to improve model performance.

One of the optimization strategies is directly replacing the original encoder with the adjusted ResNeSt-50. This method can increase the depth of the model and improve the feature extraction performance. However, since the blocks’ number of ResNeSt-50 is more than of the decoder and the ResNeSt block is deeper than the conv block, the encoder is much deeper than the decoder. The difference of depths breaks the symmetric structure of U-Net and leads to an imbalance between the encoder and decoder, which may lead to an unstable training process.

**Fig. 2:**
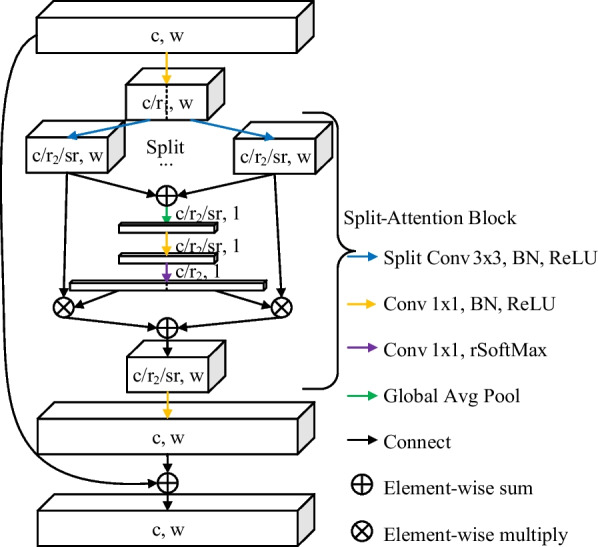
The architecture of the ResNeSt block for this task

To solve the problem, the conv blocks of encoder and decoder are equally replaced with ResNeSt blocks. The ResNeSt block is the core module of the ResNeSt series models, as shown in Fig. 2. It introduces the split-attention mechanism to the model, which improves the effect and interpretability of the model to a certain extent. In contrast to the original ResNeSt block, cardinal groups are not described in the structure diagram because there are no cardinal groups used for this task. When downsampling is performed, the pooling layer is added after the first 1 × 1 conv block and the first connection.

**Fig. 3:**
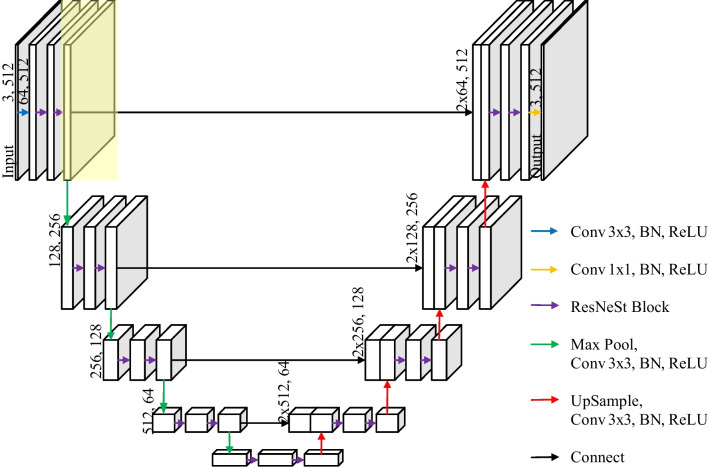
The overall architecture of SRU-Net

As shown in Fig. 3, both the encoder and decoder of U-Net use the ResNeSt block instead of the conv block to process the feature map. Meanwhile, as the yellow background shows, an additional layer is added at the top of the encoder to improve the extraction performance of the model for edges and small targets. This method improves the model's effectiveness while preserving as much of the benefits of U-Net as possible, making it simpler to backpropagate and update parameters, and enhancing the model's stability.

#### DASPP

**Fig. 4:**
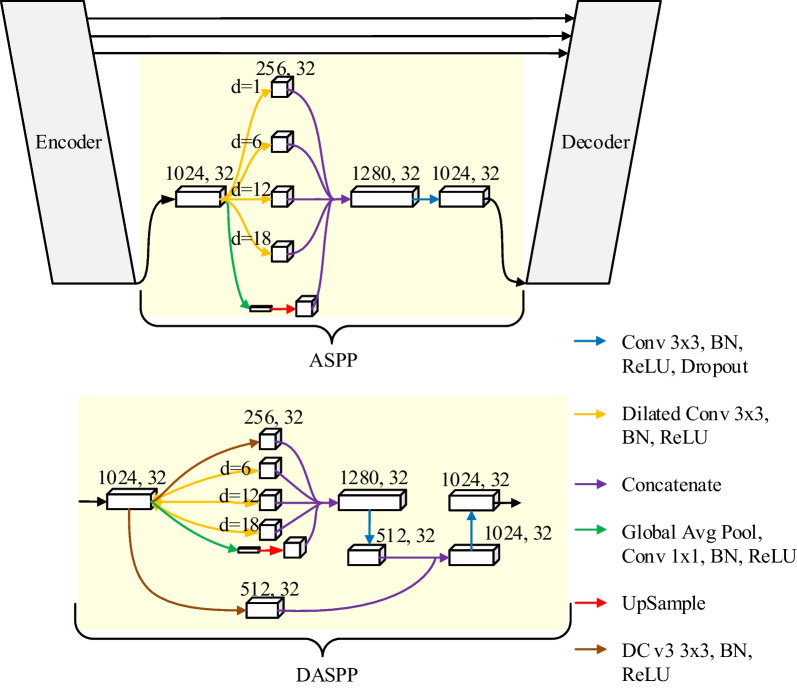
The structure of ASPP and DASPP (d is the dilated rate)

Multi-scale feature integration is a common optimization strategy for semantic segmentation, such as pyramid pooling module of the Pyramid Scene Parsing Network and the ASPP module of DeepLab v3 [36, 37]. ASPP is introduced into the U-Net structure to improve the ability of the model to combine global and local information. However, simple dilated convolution of ASPP can easily lose important features while enlarging the receptive field. To solve it, we introduce DC v3 into ASPP to design DASPP [30]. DC v3 has long-range dependencies, it allows for free selection of extraction regions, which can expand the receptive field while retaining important features [38, 39]. The structure of DASPP is shown in Fig. 4. Firstly, the dilated convolution of d = 1 in ASPP is replaced by DC v3 to suppress the loss of important features. Then a 1024 × 512 DC v3 is paralleled outside the ASPP, which adapts to extract the features of irregular shapes. Finally, the features generated by ASPP and DC v3 are concatenated and convoluted to generate multi-scale features.

### Training strategy

The weighted cross entropy loss and dice loss are combined as the loss function to evaluate the model output and guide the updating of the model parameters [40, 41]. The weighted cross entropy loss implemented by PyTorch is described as:

3 $$\begin{array}{*{20}l} {L_{{{\text{wce}}}} = - \frac{{\mathop \sum \nolimits_{n = 1}^{N} w_{{n_{tc} }} \cdot \left( {\hat{y}_{{n_{tc} }} - \log \left( {\mathop \sum \nolimits_{c = 1}^{C} \exp \left( {\hat{y}_{{n_{c} }} } \right)} \right)} \right)}}{{N \cdot \mathop \sum \nolimits_{{{\text{non}}_{c} = 1}}^{NONC} w_{{{\text{non}}_{c} }} }},} \\ \end{array}$$

where $N$ is the batch size, and $C$ is the number of classes. *w* indicates the class weight, and $\widehat{y}$ indicates the result predicted by the model. ${n}_{tc}$ represents the true class. $\mathrm{NONC}$ indicates classes, and $\sum\nolimits_{{{\text{non}}_{c} = 1}}^{NONC} {w_{{non_{c} }} }$ represents the sum of class weights that nonredundantly appeared in the batch.

The dice loss and total loss are described as:

4 $$\begin{array}{*{20}l} {L_{{{\text{dice}}}} = \frac{1}{N} \cdot \mathop \sum \limits_{n = 1}^{N} \mathop \sum \limits_{c = 1}^{C} \left( {1 - \frac{{2 \cdot y_{{n_{c} }} \cdot \hat{y}_{{n_{c} }} + \epsilon }}{{y_{{n_{c} }}^{2} + \hat{y}_{{n_{c} }}^{2} + \epsilon }}} \right),} \\ \end{array}$$

5 $$\begin{array}{*{20}l} {L_{{{\text{total}}}} = L_{{{\text{wce}}}} + L_{{{\text{dice}}}} ,} \\ \end{array}$$

where $\epsilon$ is a small number, which is 1 in this task, to prevent an exception.

To mitigate the impact of overfitting on the final result, part of the dataset is divided into the validation set to monitor the training process. The validation set is input into the model for validation output after completing each training epoch. At the end of the entire training process, the trained model with the highest dice coefficient of the validation set is used to evaluate the test set.

Samples are augmented in real time at a preset rate during the training. Data augmentation strategies include increasing and decreasing the brightness, increasing and decreasing the contract, horizontal mirroring, random angular rotation, random cropping, random stretching, etc. [42].

## Results

### Implementation details

In this experiment, 4658 samples were used as the training set and 1164 samples were used as the test set. The size of each image sent into the model was uniformly adjusted to 512 × 512. The aspect ratio of each image remained constant and the surrounding of each image was fill with 0. The training of each model was conducted over 200 epochs and with a batch size of 8. The learning rate was 0.0001, the optimizer was AdamW [43], and the model parameters were initialized with Kaiming initialization [44]. The dropout rate was uniformly 0.5 if it exists [45].

### Assessment criteria

To evaluate model performance, the specificity (SP), sensitivity (SE), precision (PR), accuracy (ACC), Intersection over Union (IoU) and dice coefficient were chosen as assessment criteria.

6 $$\begin{array}{*{20}l} {{\text{SP}} = \frac{{{\text{TN}}}}{{{\text{TN}} + {\text{FP}}}},} \\ \end{array}$$

7 $$\begin{array}{*{20}l} {{\text{SE}} = \frac{{{\text{TP}}}}{{{\text{TP}} + {\text{FN}}}},} \\ \end{array}$$

8 $$\begin{array}{*{20}l} {{\text{PR}} = \frac{{{\text{TP}}}}{{{\text{TP}} + {\text{FP}}}},} \\ \end{array}$$

9 $$\begin{array}{*{20}l} {{\text{ACC}} = \frac{{{\text{TP}} + {\text{TN}}}}{{{\text{TP}} + {\text{FP}} + {\text{TN}} + {\text{FN}}}},} \\ \end{array}$$

10 $$\begin{array}{*{20}l} {{\text{IoU}} = \frac{{{\text{TP}}}}{{{\text{TP}} + {\text{FP}} + {\text{FN}}}},} \\ \end{array}$$

11 $$\begin{array}{*{20}l} {{\text{Dice}}\;{\text{coefficient}} = \frac{{2{\text{TP}}}}{{2{\text{TP}} + {\text{FP}} + {\text{FN}}}},} \\ \end{array}$$

where TP is the number of true positives, TN is the number of true negatives, FP is the number of false positives and FN is the number of false negatives.

### Experimental result

In this experiment, FCN, U-Net, U-Net (ResNeSt-50), SRU-Net, Atrous-Pyramid Split-Attention Residual U-Net (ASRU-Net) and DSRU-Net were applied to segment thyroid glands and nodules. As a classical semantic segmentation framework, the FCN was chosen as the segmentation model for this experiment. ResNeSt, which is excellent at medical tasks, was used to improve U-Net, so the original U-Net, U-Net (ResNeSt-50) and the balanced and robust SRU-Net were tested to verify the effectiveness of the improvement strategy. Finally, ASPP/DASPP was added to U-Net to verify that capturing the contextual information on multiple scales is useful for this task.

All 6 models can perform well on this task with large data volumes and the same training strategy, and DSRU-Net works the best overall, as shown in Table 1. Although ResNeSt is a strong backbone, adding ResNeSt directly to U-Net does not significantly improve or even decreases the average dice coefficient. However, the abovementioned strategy of introducing the ResNeSt block into U-Net can significantly improve the segmentation effect. In addition, although the increase is not as large as for the ResNeSt block, ASPP can also improve the performance of U-Net, especially for thyroid nodule segmentation. Finally, DSRU-Net with better long-range dependencies contributed by DASPP achieves the best segmentation result, obtaining a 92.5% average dice coefficient and a 94.1% nodule dice coefficient.

**Table 1:** Results of 6 semantic segmentation models in this dataset

	SP (%)	SE (%)	PR (%)	ACC (%)	IoU (%)	Dice (%)	Nodule dice (%)
FCN	96.9	91.5	89.7	95.7	82.6	90.6	92.0
U-Net	96.8	91.6	90.8	95.5	84.0	91.2	92.2
U-Net (ResNeSt-50)	96.8	92.0	89.8	95.7	83.5	90.9	92.6
SRU-Net	97.4	93.3	90.1	96.6	84.7	91.7	93.5
ASRU-Net	97.6	93.4	90.3	96.8	85.1	91.8	93.5
DSRU-Net	97.9	93.8	90.8	97.2	85.8	92.5	94.1

**Fig. 5:**
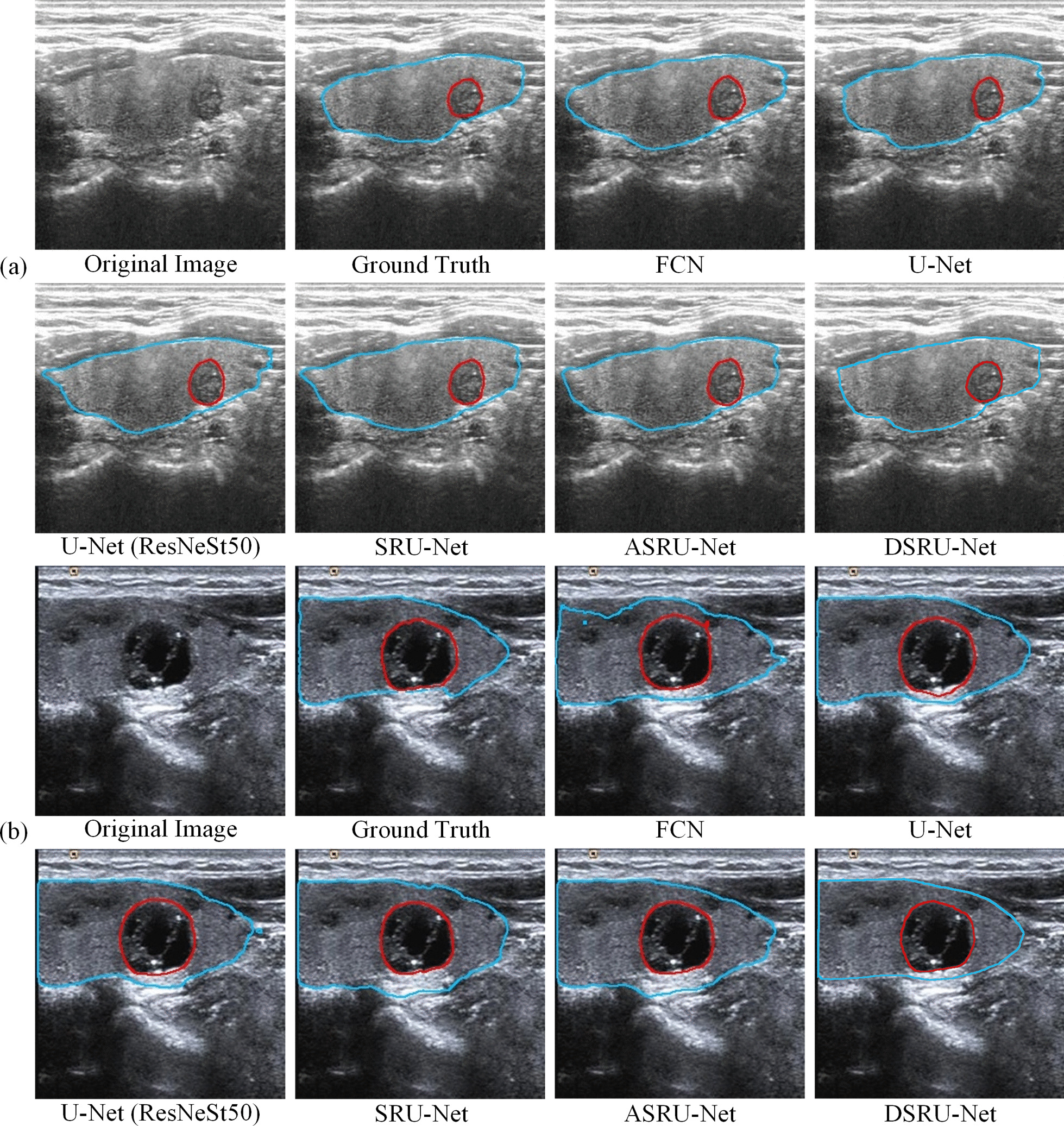
Segmentation results of 6 models for a malignant sample (**a**) and a benign sample (**b**), where the blue line outlines the gland and the red line outlines the nodule

The visual segmentation effects of 6 models for a malignant sample and a benign nodule sample are shown in Fig. 5 where blue is used to display the thyroid gland contour, and red is used to display the nodule contour. The figures show that our four improvements are generally better than traditional approaches. The figures show that the models modified by ResNeSt are better able to outline the gland and the edge of the nodules than the others. In addition, the models with ASPP/DASPP are more accurate in the segmentation of gross contours. For both sets of samples, the figures show that DSRU-Net is more sensitive than other methods, and its segmentation effect is smoother and more stable.

In addition, we evaluate positive and negative samples from AHXMU and NFH respectively with DSRU-Net, and the results are shown in Table 2. Since the training set is only from AHXMU, and the data of the two centers are different caused by various conditions such as acquisition equipment, the model performs weaker on test data from NFH than that from AHXMU. However, the difference of results between the two centers is generally acceptable, which indicates that the model has generalization ability. Possibly due to irregular shapes and calcification shadow of malignant nodules, the segmentation effect of malignant samples is lower than that of benign samples, especially the nodule dice.

**Table 2:** Results of benign and malignant samples from AHXMU and NFH with DSRU-Net

	SP (%)	SE (%)	PR (%)	ACC (%)	IoU (%)	Dice (%)	Nodule dice (%)
AHXMU_benign	99.3	95.2	92.5	98.9	88.5	93.8	95.6
AHXMU_malignant	98.9	94.8	92.5	98.4	88.1	93.6	94.1
NFH_benign	95.6	91.8	90.4	94.4	83.7	91.1	93.4
NFH_malignant	95.4	91.6	89.4	94.3	82.4	90.5	91.0

### Comparison with related research

For the purpose of demonstrating the superiority of our method, we list experimental results of thyroid segmentation literature in recent years, as shown in Table 3. These methods can be divided into 6 classes, including semi-supervised, weakly-supervised, interactive, U-Net based, DeepLab based and other methods.

**Table 3:** Results of models with various types used for thyroid segmentation in recent years

Type	Authors	Method	Year	Dataset	Noudle IoU (%)	Noudle dice (%)	Gland IoU (%)	Gland dice (%)
Semi-supervised	Kunapinun et al. [46]	StableSeg GAN	2022	Private	82.0			
Weakly-supervised	Liu et al. [47]	U2F-GAN	2021	Private		87.0		
	Yu et al. [52]	New SSE-WSSN	2022	Private	51.2	65.8		
Interactive	Shahroudnejad et al. [48]	ResDUnet	2021	Private		82.0		
	Daulatabad et al. [53]	Modified U-Net (One-Click)	2021	Private		84.0		
	Chu et al. [24]	MGU-Net	2021	Private	91.5	95.8		
U-Net based	Buda et al. [23]	U-Net (with caliper)	2020	Private		93.4		
	Liao et al. [54]	U^2^-Rnet	2021	Private	80.8	88.0	34.2	47.4
	Ataide et al. [55]	ResUNet	2021	Private	76.7	85.7		
	Ajilisa et al. [56]	Hybrid Res-UNet3	2022	DDTI	58.8	74.1		
	Lin et al. [57]	N-shape network	2022	UTNI-2021	87.0	91.9		
	Nie et al. [49]	N-Net	2022	TNUI-2021	87.2	92.0		
				DDTI	88.5	93.7		
	Li et al. [58]	BTNet	2022	Private	81.0	89.2		
				DDTI	65.4	75.7		
				BUI	73.5	81.2		
	Yadav et al. [59]	Hybrid-UNet (DsF_EPSF)	2022	DDTI + USC	86.6	93.2		
DeepLab based	Webb et al. [50]	DeepLabv3+ based convolutional LSTM	2021	Private	53.3		73.9	
	Sun et al. [60]	TNSNet	2021	Private		85.3		
Other	Ma et al. [18]	Deep CNN	2017	Private	86.8	92.2		
	Kumar et al. [51]	MPCNN	2020	Private		73.0 (64) 76.0 (78)		87.0 (68) 91.0 (80)
	Hu et al. [61]	CNN	2022	Private		83.0		
	Dai et al. [62]	SEV-Net	2022	DDTI		95.7		
	Tao et al. [63]	LCA-Net	2022	TN3K	71.2	82.1		
				TN-SCUI2020	82.7	90.3		
U-Net based		DSRU-Net (Ours)		Private	89.4	94.1	83.2	90.9

Kunapinun et al. [46] combined supervised loss and unsupervised loss to design StableSeg GAN. StableSeg GAN inhibited the instability of unsupervised GAN, improved the stability, flexibility and accuracy of segmenting thyroid nodules. Liu et al. proposed U2F‑GAN which only using bounding box as training label. It achieved a good balance between performance improvement and annotation cost by only using bounding box as training label [47]. Shahroudnejad et al. presented an interactive method named resDUnet that allows doctors to specify the region of interest. ResDUnet combined residual shortcut connections and dilated convolution on the basis of U-Net, and obtained a nodule dice coefficient of 82.0% on the authors’ private dataset [48]. Nie et al. [49] designed N-Net by introducing multi-scale input layer, attention guidance module and stackable dilated convolution block into U-Net. Their method obtained 92.0% nodule dice coefficient on TNUI-2021 and 93.7% on DDTI. Webb et al. [50] proposed DeepLabv3+ based convolutional LSTM to segment both nodules and glands. The method obtained 53.3% nodule IoU and 73.9% gland IoU on their private dataset. Kumar et al. designed MPCNN for the segmentation of nodules, glands and cystic components [51]. MPCNN achieved 73.0% nodule dice coefficient of 64 transverse images, 76.0% of 78 longitudinal images, 87.0% gland dice coefficient of 68 transverse images, and 91.0% of 80 longitudinal images.

In contrast to most methods that only segment thyroid nodules, ours segments both nodules and glandes simultaneously, which has wider clinical applications. Due to different datasets and implementation details, the advantages and drawbacks of methods can’t be directly compared. However, the experimental results of previous research also show that our method is not backward in segmentation effect, and the Dice coefficient of our method is higher than that of most other methods. However, it can be seen from the experimental results of previous studies that the dice coefficient of our method is higher than most other methods, which indicates that our method is not backward in segmentation effect.

## Discussion

In this paper, we propose DSRU-Net for the thyroid segmentation task. U-Net is a popular model with FCN architecture for medical images. U-Net's skip-connection structure effectively alleviates the information loss when the decoder is upsampled and helps restore high-resolution spatial content [28]. However, for this task, U-Net falls slightly short in two respects. One is that the method is insufficient at extracting the high-resolution information from the shallow layer, and the other is that the method lacks the ability to analyze the internal relationship of low-resolution information from the deep layer.

**Fig. 6:**
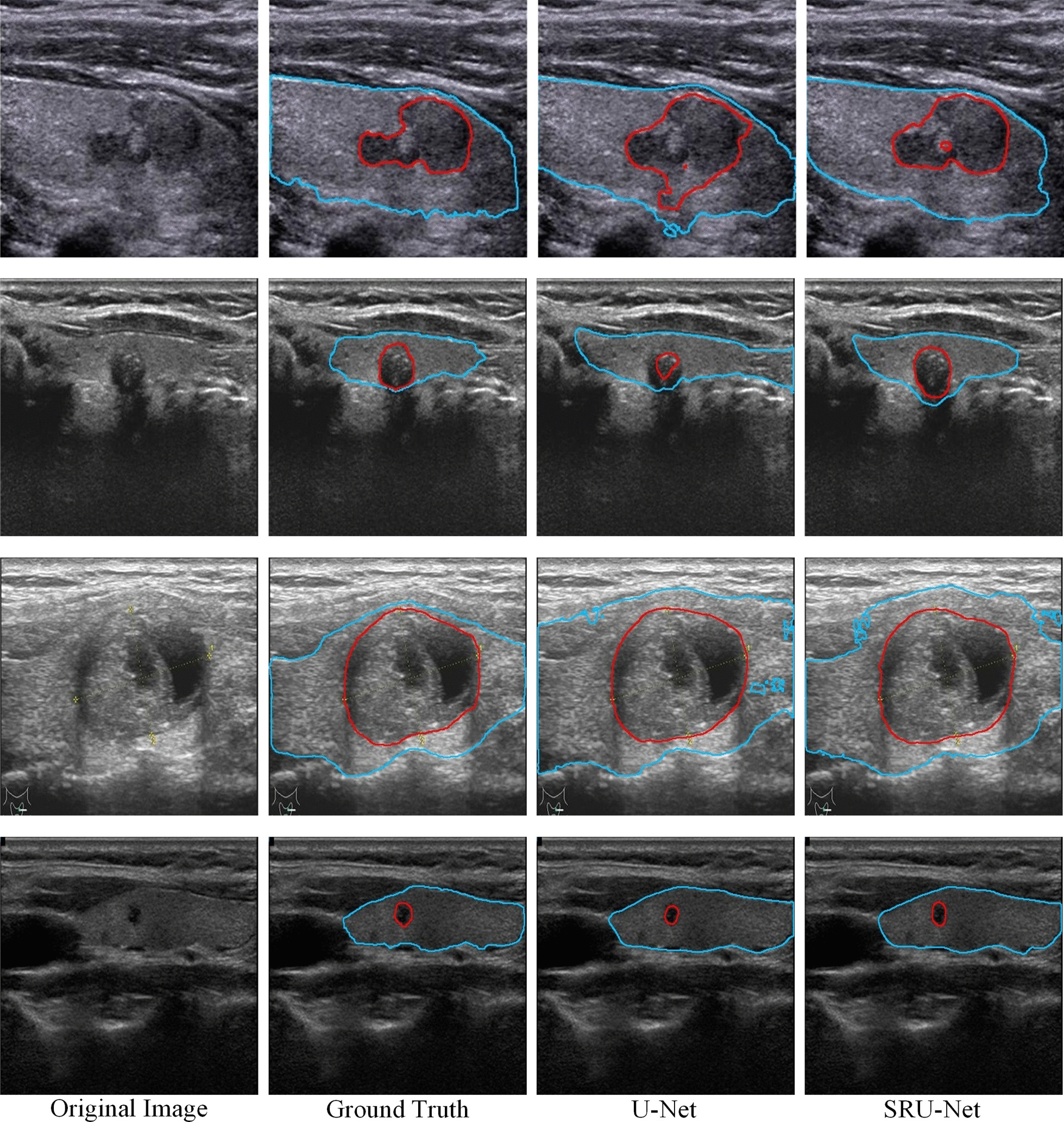
Results of samples segmented by models with and without the ResNeSt block, where the blue line outlines the gland and the red line outlines the nodule

U-Net’s poor extraction of high-resolution information will lead to its weak extraction of small targets and edges, which can be mitigated by the introduced ResNeSt block. The segmentation of edges and small targets plays a critical role in this task. As shown in Fig. 6, due to the invasiveness of nodules, acoustic shadows, and poor quality images collected by old ultrasonic devices, the segmentation of some image edges is challenging. In addition, there are some small nodules in some images, and their segmentation effect will be limited by a shallow high-resolution information extraction layer. However, the structure of the original U-Net makes it slightly insufficient at handling these issues. For example, the feature tensor with a height and width of 568 (512 in this task with padding) output from the original U-Net encoder is processed only by two convolution layers. However, a ResNeSt block performs two ordinary 1 × 1 convolutions, one 3 × 3 group convolution on the feature tensor, and two 1 × 1 convolutions on global information. In SRU-Net, for the first feature tensor output by the encoder, even if the four convolution layers of the attention tensor are ignored, there are seven convolution layers for processing. Compared to U-Net with only two convolution layers in the front, our method effectively increases the depth of the shallow layer and ensures that high-resolution features can also be fully extracted. Moreover, since ResNeSt inherits the residual structure of ResNet, it can alleviate the gradient disappearance of deep layers [64]. As shown in Fig. 6, SRU-Net performs better than U-Net in these situations.

**Fig. 7:**
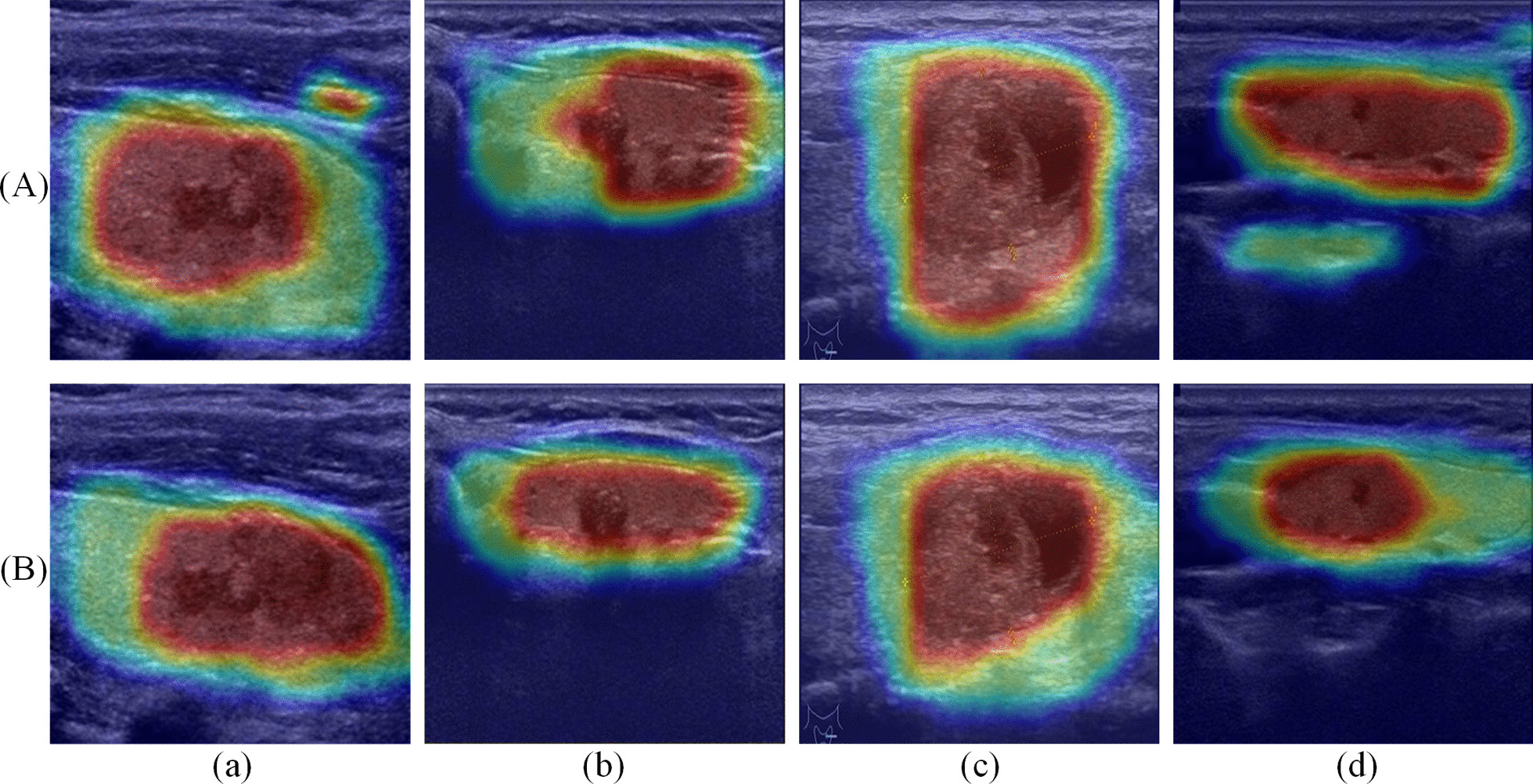
Activation visualization of **A** U-Net and **B** SRU-Net

To further illustrate the effectiveness and reliability of the introduction of ResNeSt blocks, heat maps are used to visualize U-Net and SRU-Net activation. We respectively input the four ultrasonic images in Fig. 6 into U-Net and SRU-Net, average and normalize the features output by the encoders, and enlarge the features to the size of the original images to generate heat maps. The visualization results are shown in Fig. 7, it can be seen that the two models mainly focus on thyroid glands and nodules. Among them, U-Net's attention is relatively scattered, especially for samples (a) and (d), U-Net is interested in irrelevant information around. In contrast, SRU-Net pays more attention to the features of important regions, has stronger anti-interference ability to irrelevant information, and shows higher reliability.

**Fig. 8:**
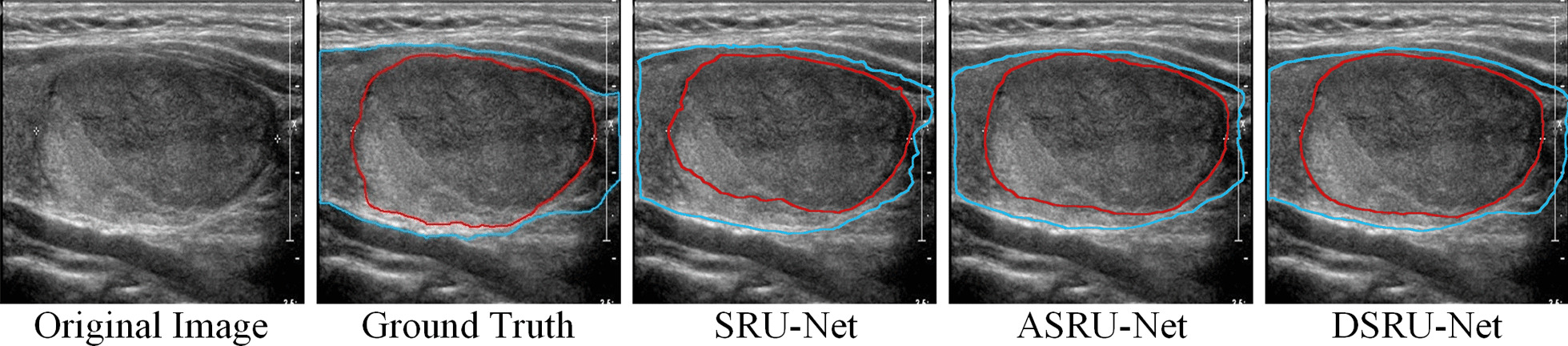
Results of super large nodule sample segmented by SRU-Net, ASRU-Net which contains ASPP and DSRU-Net which contains DASPP, where the blue line outlines the gland and the red line outlines the nodule

DASPP can improve the segmentation performance by making better use of deep features especially for large targets. For the original U-Net, the features extracted by the decoder lack global information. After convolutions and max pooling, each element of the encoder's innermost feature tensor contains at most information about the 140 × 140 region of the input image. However, the size of thyroid glands and some super-large nodules may be larger than 140 × 140, and the feature matrix lacking global information may be slightly inadequate to represent them. DASPP uses global average pooling to provide global information while using different dilation rates of dilated convolutions to extract context features at multiple scales. It not only allows each element of the feature matrix to understand the complete information of glands and nodules, but also obtains local information at different scales. The DC v3 of DASPP inhibits the interference of irrelevant information, makes it more suitable for target regions with different shapes, and has better detection ability for target edges and targets with special shapes. As shown in Fig. 8, DSRU-Net containing DASPP is more complete in segmenting large nodules than U-Net. Meanwhile, DSRU-Net has better segmentation effect on target edge than ASRU-Net containing ASPP.

**Fig. 9:**
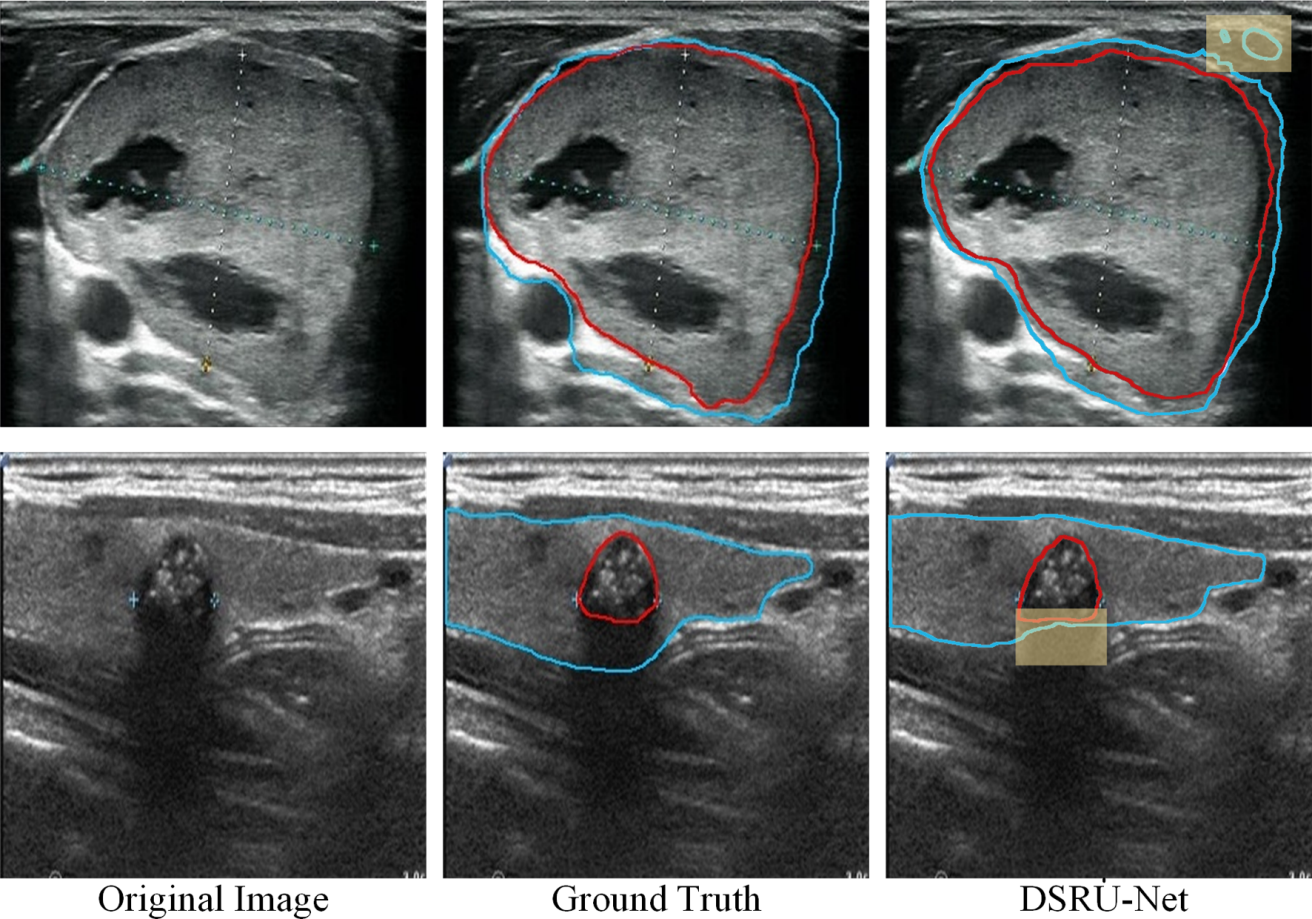
Unsatisfactory examples segmented by DSRU-Net, where the blue line outlines the gland and the red line outlines the nodule

There may be room for further optimization and limitations in our method. For example, Zhou et al. [41] proposed an improved U-Net model called U-Net++. U-Net++ provides useful suggestions for improving segmentation performance by redesigning skip connections and making networks more lightweight with model pruning, and the model performs well on electron microscopy and cell datasets, among others. We intend to apply U-Net++ to our dataset in the early stage of the experiment, but its performance is inferior compared with that of U-Net on our dataset. But to improve U-Net, redesigning skip connections is a great idea, and the proper strategies might have the best results. In addition, our method is not ideal for segmentation of certain glands, as shown in Fig. 9. One problem is that multiple glands may be segmented. However, since the thyroid gland is generally only a continuous piece, the area can be calculated by regional connectivity so as to filter out smaller noise areas to alleviate this problem. Another problem is that for some areas with low echo or no echo at the edge of the glands, our method may regard them as external areas of the glands. This may be mitigated by data augmentation such as increasing and decreasing contrast in random regions.

## Conclusion and outlook

We present an improved U-Net architecture called DSRU-Net with ResNeSt block, ASPP and DC v3 for thyroid gland and nodule segmentation. DSRU-Net is demonstrated to be effective by comparing segmentation findings from the original method and several improved versions using the dice coefficient and other assessment criteria. The comparison with related research shows that the method is advanced. The method can better concentrate on crucial information, according to activity visualization. As shown in visualization segmentation results, DSRU-Net cannot only reasonably analyze shallow features to better segment edges and small nodules, but also analyze deep information at multiple scales to improve the segmentation effect of glands and super-large nodules.

Although our method can also cope with harsh situations like acoustic shadows, there are still numerous techniques that can be used to further enhance our method's robustness. One solution is to select challenging images and ordinary images and then use GAN to augment challenging images and randomly interfere with common images to make a separate dataset. The training strategy of using this dataset and the original dataset alternately may be an effective optimization strategy. However, the inference process of our model is slow. Subsequently, we can compare the effects of reducing the number of downsampling operations or the number of convolution kernels on model performance and design a more efficient model without losing accuracy.

## Data Availability

The data that support the findings of this study are available on request from the corresponding author on reasonable request. The data are not publicly available due to privacy or ethical restrictions.

## Abbreviations

AHXMU: Affiliated Hospital of Xuzhou Medical University
NFH: Nanjing First Hospital
ASPP: Atrous spatial pyramid pooling
SVM: Support vector machine
CNN: Convolutional neural network
SP: Specificity
SE: Sensitivity
DSRU-Net: Deformable-pyramid split-attention residual U-Net
DC: Deformable convolution
IoU: Intersection over Union
US: Ultrasound
SRU-Net: Split-attention residual U-Net
ASRU-Net: Atrous-pyramid split-attention residual U-Net
